# An effective oxidation approach for luminescence enhancement in CdS quantum dots by H_2_O_2_

**DOI:** 10.1186/1556-276X-7-672

**Published:** 2012-12-12

**Authors:** Woojin Lee, Hoechang Kim, Dae-Ryong Jung, Jongmin Kim, Changwoo Nahm, Junhee Lee, Suji Kang, Byungho Lee, Byungwoo Park

**Affiliations:** 1 WCU Hybrid Materials Program, Department of Materials Science and Engineering, Research Institute of Advanced Materials, Seoul National University, Seoul, 151-744, Korea

**Keywords:** Photoluminescence, Surface passivation, Quantum efficiency

## Abstract

The effects of surface passivation on the photoluminescence (PL) properties of CdS nanoparticles oxidized by straightforward H_2_O_2_ injection were examined. Compared to pristine cadmium sulfide nanocrystals (quantum efficiency ≅ 0.1%), the surface-passivated CdS nanoparticles showed significantly enhanced luminescence properties (quantum efficiency ≅ 20%). The surface passivation by H_2_O_2_ injection was characterized using X-ray photoelectron spectroscopy, X-ray diffraction, and time-resolved PL. The photoluminescence enhancement is due to the two-order increase in the radiative recombination rate by the sulfate passivation layer.

## Background

Semiconductor nanocrystals or quantum dots have attracted great attention because their optical and electrical properties can be tuned by changing their sizes and surface states [1-6]. Photoluminescence (PL) characteristics of semiconductor nanocrystals are strongly dependent on their surface states since a large portion of atoms are located at or near the surface of nanoparticles, forming dangling bonds as main trap states against radiative recombination. Focused on the surface states, various strategies for the enhancement of optical properties in CdS nanocrystals have been developed by employing a core/shell structure, size-selective photoetching, and surface passivation by reducing agents [7-12].

In this regard, the artificial formation of an oxide layer on the surface of CdS nanocrystals holds great potential for surface passivation and tuning the size of nanocrystals [13]. Despite the aforementioned advantages, the formation of an oxide layer leads to the elimination of the passivating ligands bound to the surface of quantum dots. It is difficult to synthesize surface-oxidized quantum dots with ligands by traditional methods [14,15].

In this study, we have developed a facile and straightforward oxidation process by injection of H_2_O_2_ with subsequent ligand exchange for highly luminescent CdS quantum dots. In order to describe the mechanism of PL enhancement, the changes in the chemical states during the oxidation process are examined based on X-ray photoelectron spectroscopy (XPS) data. The correlations of the enhanced PL properties with the quantum dot size, local strain, chemical states, and radiative recombination rates are systematically investigated.

## Methods

The CdS nanocrystals were synthesized using a reverse micelle method previously reported by Wang et al. [16]. Cadmium chloride (CdCl_2_, 0.182 g) and sodium sulfide (Na_2_S, 0.078 g) were dissolved separately in distilled water (15 mL) and stirred until complete dissolution. The cadmium chloride solution was placed into an autoclave followed by the addition of sodium sulfide. Linoleic acid ((C_17_H_31_)COOH, 2.4 mL) and sodium linoleate ((C_17_H_31_)COONa, 2 g) dissolved in ethanol were added to the resulting solution. The resultant CdS nanocrystals were precipitated using centrifugation and cleaned several times with ethanol. After synthesis, the CdS nanocrystals were dispersed into chloroform (CHCl_3_, 40 mL), which displayed a transparent yellow color.

For the oxidation step of CdS nanocrystals, 3.0 wt. % H_2_O_2_ solution was added to the solution of nanocrystals in a dark environment, and *n*-butylamine ((C_4_H_9_)NH_2_, 10 mL) was added for the ligand exchange. The samples were oxidized with the addition of different amounts (0, 0.8, 1.2, 1.6, 2.0, 2.4, and 2.8 mL) of H_2_O_2_ solution. During injection, 0.4 mL of the H_2_O_2_ solution was repeatedly injected at 24-h time intervals.

The nanostructure of the CdS nanoparticles was analyzed by X-ray diffraction (XRD, M18XHF-SRA, MAC Science Co., Yokohama, Japan). The PL spectra were measured using a spectrofluorometer (FP-6500, JASCO, Essex, UK) with a Xe lamp, and the absorption spectra were recorded on a UV/Vis spectrophotometer (Lambda 20, PerkinElmer, Waltham, MA, USA). The quantum efficiency of colloidal CdS samples was estimated using Rhodamine 6G in ethanol (quantum efficiency of approximately 95% for an excitation wavelength of 488 nm) by comparing their absorbance in order to examine the luminescence properties quantitatively [17]. The surface chemical states of CdS nanocrystals were analyzed by XPS (Sigma Probe, Thermo VG Scientific, Logan, UT, USA) using Al *Kα* radiation (1,486.6 eV).

## Results and discussion

**Figure 1:**
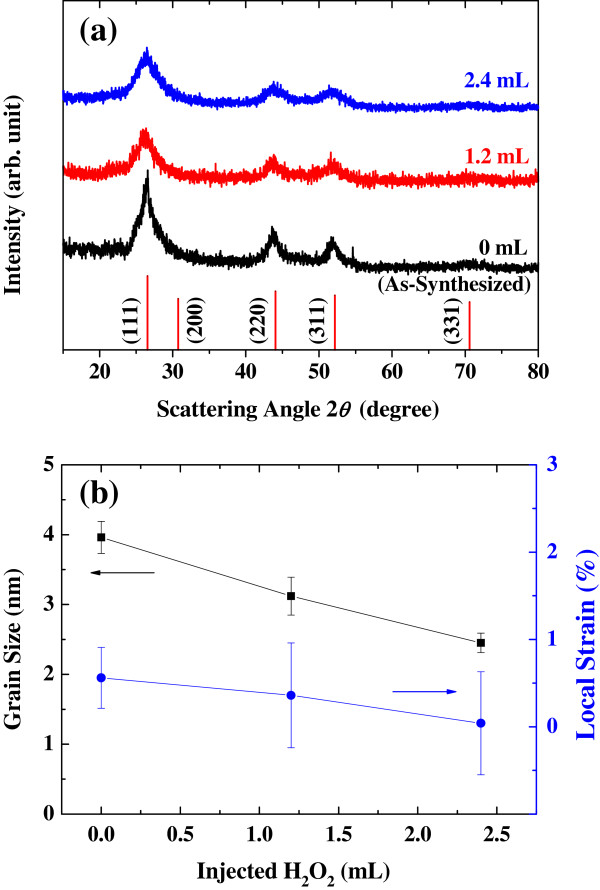
**XRD patterns, nanocrystal size, and local strain.** (**a**) X-ray diffraction of CdS nanocrystals and (**b**) the size of nanocrystals and their local strain. The peak positions and intensities of CdS (JCPDS #75-0581) are marked.

The effects of oxidation on the size and strain of CdS nanocrystals were investigated by XRD analysis. Figure 1 shows the XRD patterns of CdS nanocrystals prepared with different oxidation steps. To qualitatively estimate the local strain and the effective size of CdS nanocrystals, the diffraction peak widths (full width at half maximum) were fitted with the scattering vector (*k* = (4*π*/*λ*)sin*θ*) using a double-peak Lorentzian function, considering the effect of *Kα*_1_ and *Kα*_2_[18-21] and the instrumental broadening effect.

As shown in Figure 1b, the core size of CdS nanocrystals gradually decreases with the increasing amount of H_2_O_2_ solution, indicating the formation of an oxide layer. As the thickness of the oxide layers increases, the local strain of CdS nanocrystals slightly decreases. The synthesis of nanocrystals at room temperature can lead to defective shells of CdS quantum dots due to the insufficient kinetics for complete crystallization [22-24]. Therefore, the reduced local strain may be caused by the oxidation of this defective shell by H_2_O_2_.

**Figure 2:**
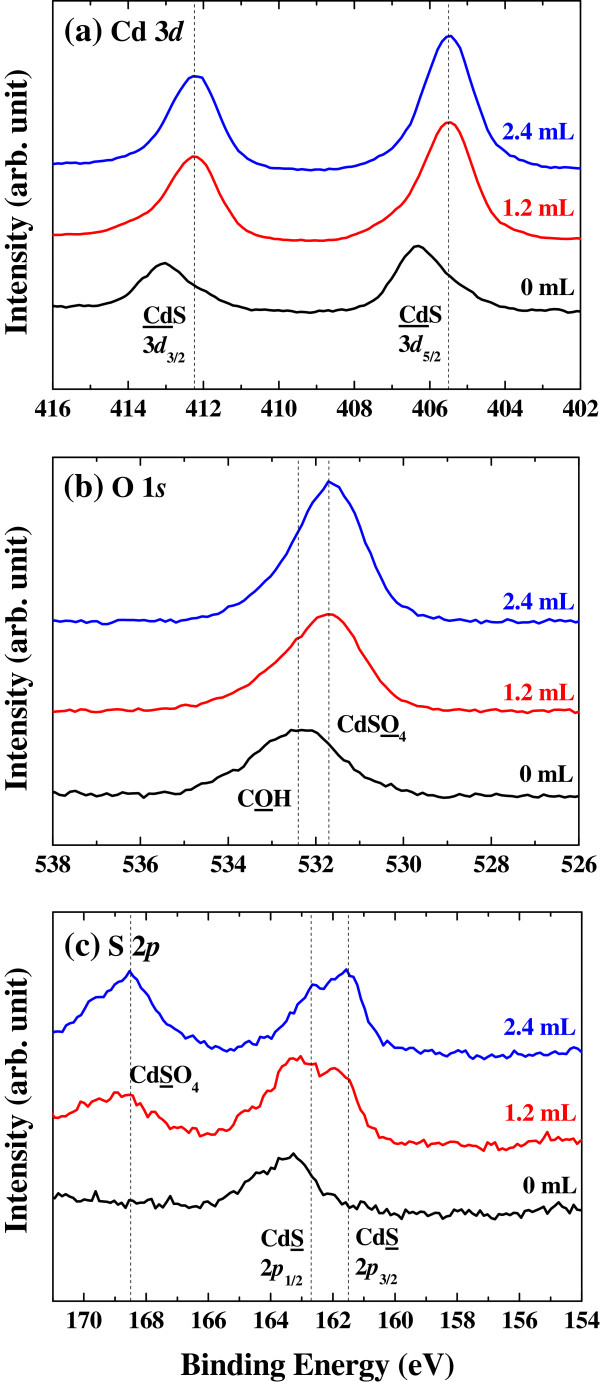
**XPS spectra.** The spectra correspond to (**a**) Cd 3*d*, (**b**) O 1 *s*, and (**c**) S 2*p* for the CdS nanocrystals with various amounts of injected H_2_O_2_ solution. The dashed lines on each spectrum are from [26] and [27].

**Figure 3:**
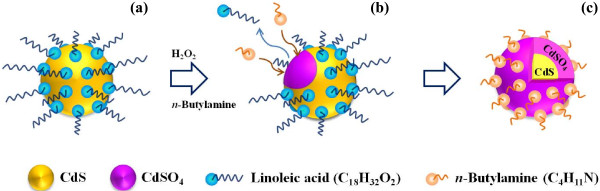
**Schematic figure of the oxidation process and surface modification with *n*-butylamine.** (**a**) The synthesized nanocrystal covered with linoleic acid, (**b**) loss of organic ligands during surface oxidation, and (**c**) surface modification with *n*-butylamine.

The change in surface states of CdS nanocrystals after oxidation was investigated by XPS (Figure 2). The peak shift in O 1*s* from 532.5 to 531.7 eV indicates the change of chemical bonding from carboxyl acid bound to nanocrystals to cadmium-containing oxide [25-27]. In addition, the 168.5 eV peak from S 2*p* and the 531.7 eV peak from O 1*s* are observed only after oxidation [26], indicating to the formation of CdSO_4_ layers. A proposed mechanism for the surface oxidation in CdS nanocrystals is schematically described in Figure 3. During the oxidation reaction with H_2_O_2_, organic ligands dissolve into the solvent [28], and the surface modification by the amine functional group prevents the quantum dots from agglomeration [29].

**Figure 4:**
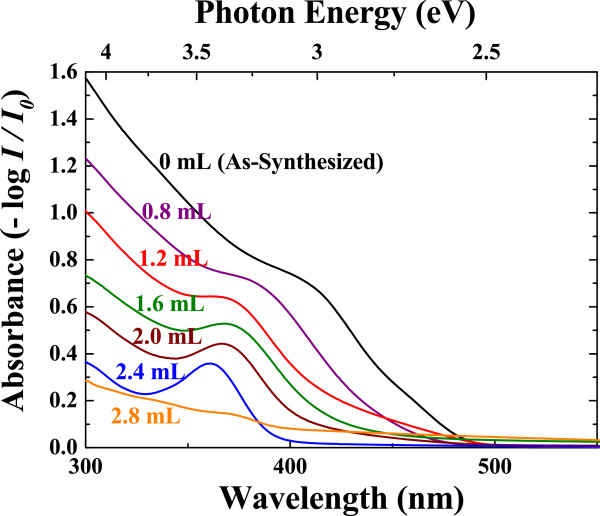
**The absorbance spectra of CdS nanoparticles.** As the amount of H_2_O_2_ solution increased, the optical bandgap and exciton peak shifted to a higher energy.

As the amount of H_2_O_2_ solution increases, the absorbance spectra of the samples exhibit blueshift depending on their size reduction, and the first exciton peak becomes clear with the addition of H_2_O_2_ solution over 1.6 mL (Figure 4). The exciton peak with 2.8 mL of H_2_O_2_ injection is not clearly observed, which may have resulted from the nearly complete oxidation to the core part of quantum dots due to fast oxidation by H_2_O_2_[24].

**Figure 5:**
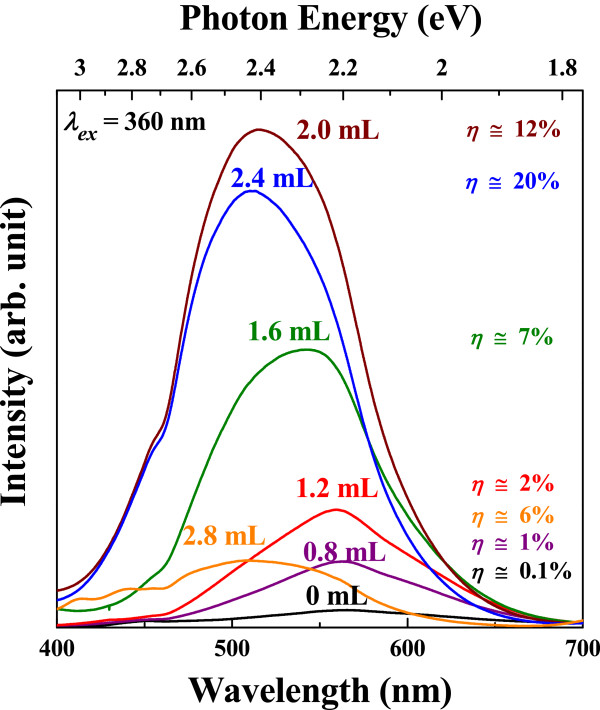
**Photoluminescence spectra of CdS nanoparticles with various amount of H**_**2**_**O**_**2**_**.**

The luminescence characteristics display broad emission ranging from 450 to 650 nm, which originates from the trap-state emission, as shown in Figure 5[11,12]. The highest emission peak intensity of CdS nanocrystals is about two orders of magnitude higher than that of the as-synthesized one. Moreover, the CdS quantum dots oxidized with the addition of H_2_O_2_ solution over 1.6 mL show a weak band-edge emission at 450 nm and spectral blueshift of the photoluminescence.

**Figure 6:**
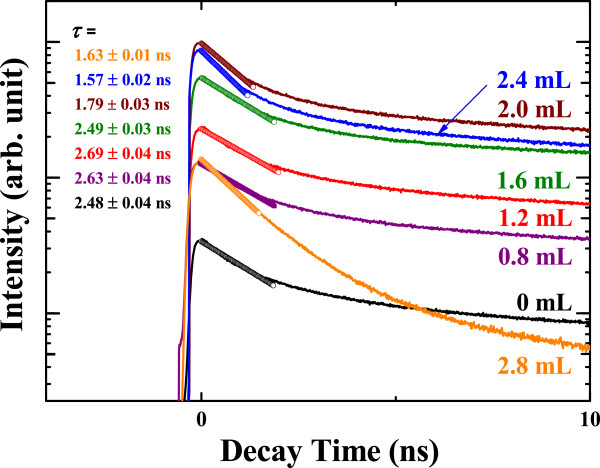
**Decay curves of CdS nanoparticles as a function of H**_**2**_**O**_**2**_**solution with corresponding decay time (*τ*).**

**Figure 7:**
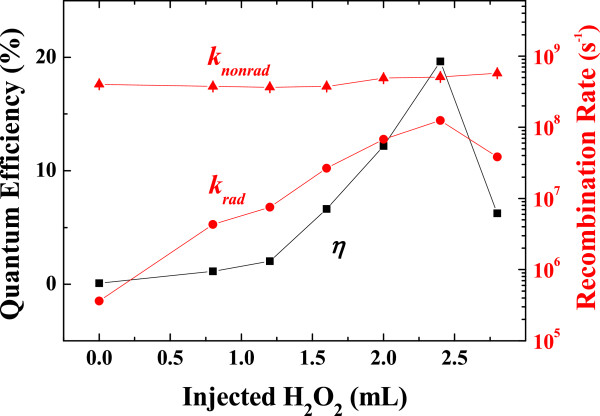
**Quantum efficiency, radiative recombination rate, and nonradiative recombination rate as a function of H**_**2**_**O**_**2**_**solution.**

For the carrier dynamics in oxidized CdS nanocrystals, each decay time (*τ* = *k*_total_^-1^) was acquired from a single-exponential fitting at the initial stage of the time-resolved PL (Figure 6). The quantum efficiency (*η*) is

(1) $$\eta =\frac{k_{\mathrm{rad}}}{k_{\mathrm{rad}}+k_{\mathrm{nonrad}}}=k_{\mathrm{rad}}\times \tau$$

where *k*_total_, *k*_rad_, *k*_nonrad_, and *τ* are the total, radiative, and nonradiative recombination rates, and the decay time (*k*_rad_ + *k*_nonrad_)^−1^, respectively. Figure 7 shows the quantum efficiency and radiative/nonradiative recombination rates of oxidized nanocrystals with different amounts of injected H_2_O_2_ solution. The quantum efficiency enhancement in CdS is mainly caused by the increased radiative recombination rate, while the nonradiative recombination rate remains constant, even though our previous papers reported reduced nonradiative recombination by the formation of a passivation layer on quantum dots [30,31].

## Conclusions

Highly luminescent CdS QDs were obtained using a facile and straightforward H_2_O_2_ oxidation process with ligand exchange. The amount of H_2_O_2_ used in the CdS oxidation process was correlated with the quantum dot size, local strain, chemical states, and radiative/nonradiative recombination rates. The oxidized CdS nanocrystals exhibited a quantum efficiency (20%) two orders of magnitude higher than that of an as-synthesized sample (0.1%) by an effective passivation promoting radiative recombination rate.

## Competing interests

The authors declare that they have no competing interests.

## Authors’ contributions

WL and DRJ drafted and revised the manuscript. HK carried out the synthetic experiments and characterizations. JK, CN, JL, SK, and BL participated in the scientific flow. BP conceived the study and participated in its design and coordination. All authors read and approved the final manuscript.
